# Hippocampal Representation of Touch-Guided Behavior in Rats: Persistent and Independent Traces of Stimulus and Reward Location

**DOI:** 10.1371/journal.pone.0016462

**Published:** 2011-01-28

**Authors:** Pavel M. Itskov, Ekaterina Vinnik, Mathew E. Diamond

**Affiliations:** Sector of Cognitive Neuroscience, Scuola Internazionale Superiore di Studi Avanzati (SISSA), Trieste, Italy; University of New South Wales, Australia

## Abstract

Understanding the mechanisms by which sensory experiences are stored remains a compelling challenge for neuroscience. Previous work has described how the activity of neurons in the sensory cortex allows rats to discriminate the physical features of an object contacted with their whiskers. But to date there is no evidence about how neurons represent the behavioural significance of tactile stimuli, or how they are encoded in memory. To investigate these issues, we recorded single-unit firing and local field potentials from the CA1 region of hippocampus while rats performed a task in which tactile stimuli specified reward location. On each trial the rat touched a textured plate with its whiskers, and then turned towards the Left or Right water spout. Two textures were associated with each reward location. To determine the influence of the rat's position on sensory coding, we placed it on a second platform in the same room where it performed the identical texture discrimination task. Over 25 percent of the sampled neurons encoded texture identity – their firing differed for two stimuli associated with the same reward location – and over 50 percent of neurons encoded the reward location with which the stimuli were associated. The neuronal population carried texture and reward location signals continuously, from the moment of stimulus contact until the end of reward collection. The set of neurons discriminating between one texture pair was found to be independent of, and partially overlapping, the set of neurons encoding the discrimination between a different texture pair. In a given neuron, the presence of a tactile signal was uncorrelated with the presence, magnitude, or timing of reward location signals. These experiments indicate that neurons in CA1 form a texture representation independently of the action the stimulus is associated with and retain the stimulus representation through reward collection.

## Introduction

Whisker-mediated tactile perception in rats has been the object of intense investigation[1]–[9]. In spite of progress in understanding the coding of physical features, little is known about how tactile events are encoded in memory and how neurons represent the meaning of tactile stimuli in the context of behavior.

To address these issues, we have investigated the CA1 region of hippocampus. Hippocampus has been posited to be crucial for bridging across events that are causally linked, but separated in time[10], [11]. Our hypothesis was that it would hold short-term traces of salient sensory signals – those used to guide behavior – until reward collection. Since hippocampus contains a prominent representation of space[12], [13], we could expect to uncover tactile signals only after teasing them apart from those related to the animal's head and body movement through space. We isolated tactile signals by presenting 3 or 4 textures, 2 of which were associated with one reward location; because two stimuli were always associated with the identical action and reward location, any difference in activity must reflect the coding of touch rather than some aspect of explicit behavior.

This approach allowed us to distinguish tactile signals and track their evolution over the trial time course; we also tracked the neuronal activity related to the animals' actions. Finally, we explored the interaction of different signals by asking to what extent the population of neurons recruited in the representation of touch overlapped, or was distinct from, the population of neurons recruited in the representation of reward location.

## Results

### Touch-guided Behavior

**Figure 1:**
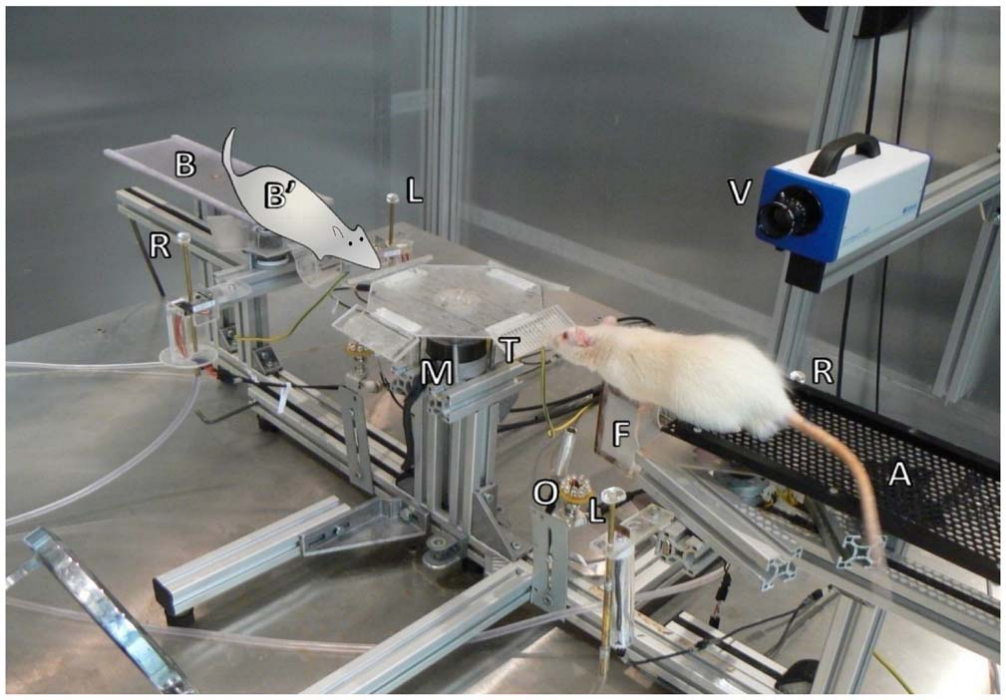
Experimental setup. Photograph of texture palpation taken under visible light. A-platform A, B-platform B, B'-rat position on platform B, M-motor for automated presentation of textures, T-textured plate, F-foot rest, O-optical triggers for touch detection, L, R-left and right water spouts, V-high-speed video camera. While the rat collected the reward, the motor rotated to put one of the four textures, in random order, in position for the next trial. The motorized foot rest (F) was then extended to allow the rat to reach the texture. When the rat withdrew after texture palpation, the foot rest was retracted to prevent the animal from returning to the texture. Optical triggers were positioned in front of the texture to detect touch and the beginning of turn (i.e. the moment when the animal retracted from the texture). The animal's choice was detected by another set of optical triggers (not labeled) located immediately adjacent to the drinking wells.

**Figure 2:**
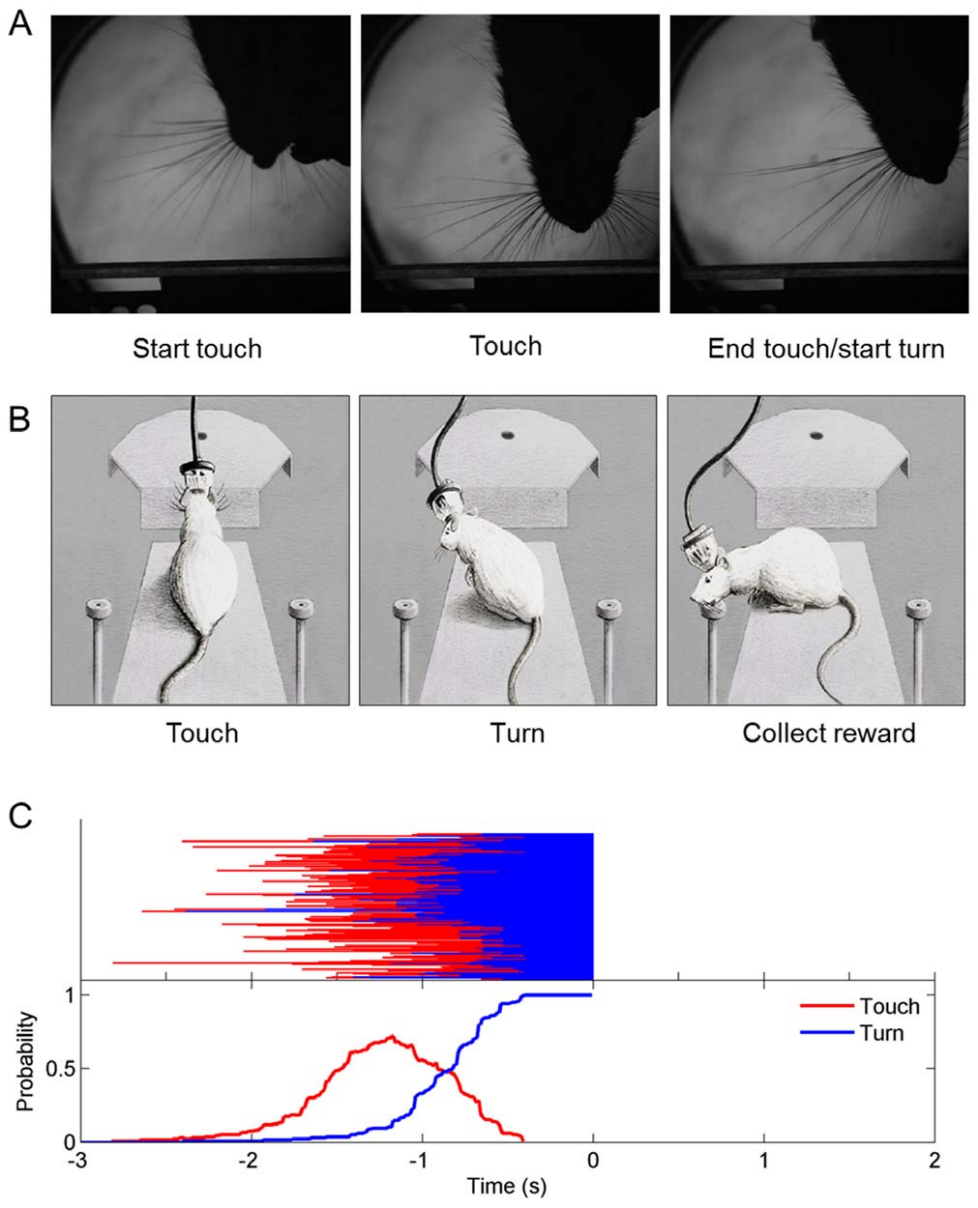
Time course of behavior. (A) Images from overhead high-speed video illustrating the initial contact (left), palpation of the texture (middle) and the instant of withdrawal (right), equivalent to the beginning of the turn. (B) On each trial the rat approached the plate, triggering an optic sensory, and touched the texture with its whiskers (left). Once the animal identified the texture, it turned to the right or to the left drinking spout (middle) where it collected the water reward (right). (C) Time course of behavior in a typical recording session. Upper plot: for all the trials, horizontal red lines indicate the duration of touch time and blue lines indicate turn time. Trials are aligned on reward onset, 0 ms. Lower plot: By summating over trials, we calculated the probability that the rat was engaged in touch (red) or turn (blue) at any given time.

This study set out to identify how neurons in rat hippocampus represent tactile stimuli and the behavior associated with those stimuli. Animals perched on one of two platforms from which they could contact, with their whiskers, a plate mounted on a central motor (Figure 1; Video S1). Textures are described in Materials and Methods. They discriminated the surface of the plate and, according to its identity, turned left or right to obtain a reward (Figure 2).

Four rats (rats 1–4) were trained and tested on only one platform (platform A), the one-platform task. Three textures were used (T1–T3): two textures were associated with one reward location and the remaining texture with the opposite reward location. Associations between texture and reward location were fixed for each animal but were varied across rats (Table S1). Two rats (rats 5, 6) were trained and tested on platforms A and B, the two-platform task. Four textures were used (T1–T4): two textures were associated with each reward location. Training began on just one platform (rat 5 – platform A; rat 6 – platform B). After reaching a stable performance of at least 75 percent correct per session, the animals were exposed to the second platform. The same set of textures was presented on the two platforms (Figure 1); the association between texture and reward location remained constant in egocentric coordinates. Unexpectedly, neither rat showed immediate transfer of knowledge from the first to the second platform. About 3–5 days (about 400 trials) of additional training were required for the animals to relearn the task. For the duration of the experiment, they showed lower performance in the second location (median performance across all recording sessions: 90.2 percent on first platform, 80.1 percent on second platform; Wilcoxon sign rank test, p<0.00001). The lack of immediate transfer suggests they formed two independent representations of the task; at the second location, the texture-North/South (allocentric) association competed with the texture-left/right (egocentric) association, with the allocentric association initially stronger than the egocentric one[14]. Physiological correlates of this independence will be presented later.

### Timing of Behavioral Events

The sequence of actions in well-trained rats was stereotypical (Figure 2). We verified that the durations of the events (touch, turn) leading to reward collection did not differ for the two textures associated with the same reward location. They touched the texture for just over 500 ms (median 530 ms, interquartile range 375–766 ms). The turn duration (time elapsed between withdrawal from the texture and triggering of the optical sensor at the water spout) was about 800 ms (median 812 ms, interquartile range 656–1032 ms). To analyze neuronal activity, spike trains were aligned to the instant when the optical sensor at the drinking well was interrupted, referred to as “reward onset” and set to 0 ms.

### Neuronal Representation of Texture

The experiment was designed to make encoding of texture identity explicit. Since the observed behavior did not differ for the texture pair associated with one reward location, any differences in neuronal response could be attributed to texture rather than the trajectory or spatial coordinates of the animal's head or body. Although neuronal firing likely discriminated between textures associated with disparate reward locations, we limited the texture analysis to pairs of textures associated with the same reward location in order to avoid any entanglement with reward location signals.

We recorded 896 neurons in six rats (490 pyramidal cells and 396 interneurons; Figures S1–S2). All reported results are from correct trials (however see Figure S3). To quantify texture coding, we measured the information carried by neuronal firing rate in a 400 ms window that was advanced from −2,000 ms to +2,000 ms, relative to reward onset, in 25 ms steps. In order to estimate the significance of the texture coding in each neuron, texture information was averaged over time and compared to the average values of information obtained by shuffling texture labels across trials (p<0.05 see Materials and Methods).

To measure the overall strength of the texture signal in the total population of neurons, we conducted the following test. We examined each neuron for the presence of a significant texture signal, as above, under each possible condition (that is, for all reward locations). This provided an observed number of texture signals within the data set. Then for each neuron, we shuffled the texture labels and examined the neuron again for the presence of a significant (p<0.05) texture signal. This provided the number of texture signals that could be expected by chance, given that the texture labels had been shuffled. We repeated the shuffling and recounting procedure 200 times to produce a distribution of the number of texture signals expected by chance. Finally, we compared the actual number of texture signals (with texture labels intact) to the distribution of numbers of texture signals expected by chance. The result is given in Figure S4. By comparison to the distribution of counts obtained after shuffling, it can be calculated that the probability of the observed number of texture signals occurring by chance is p<0.0000000005. Thus, the population on the whole carried a strong texture message. On that basis, we hereafter consider the signal in individual neurons.

**Figure 3:**
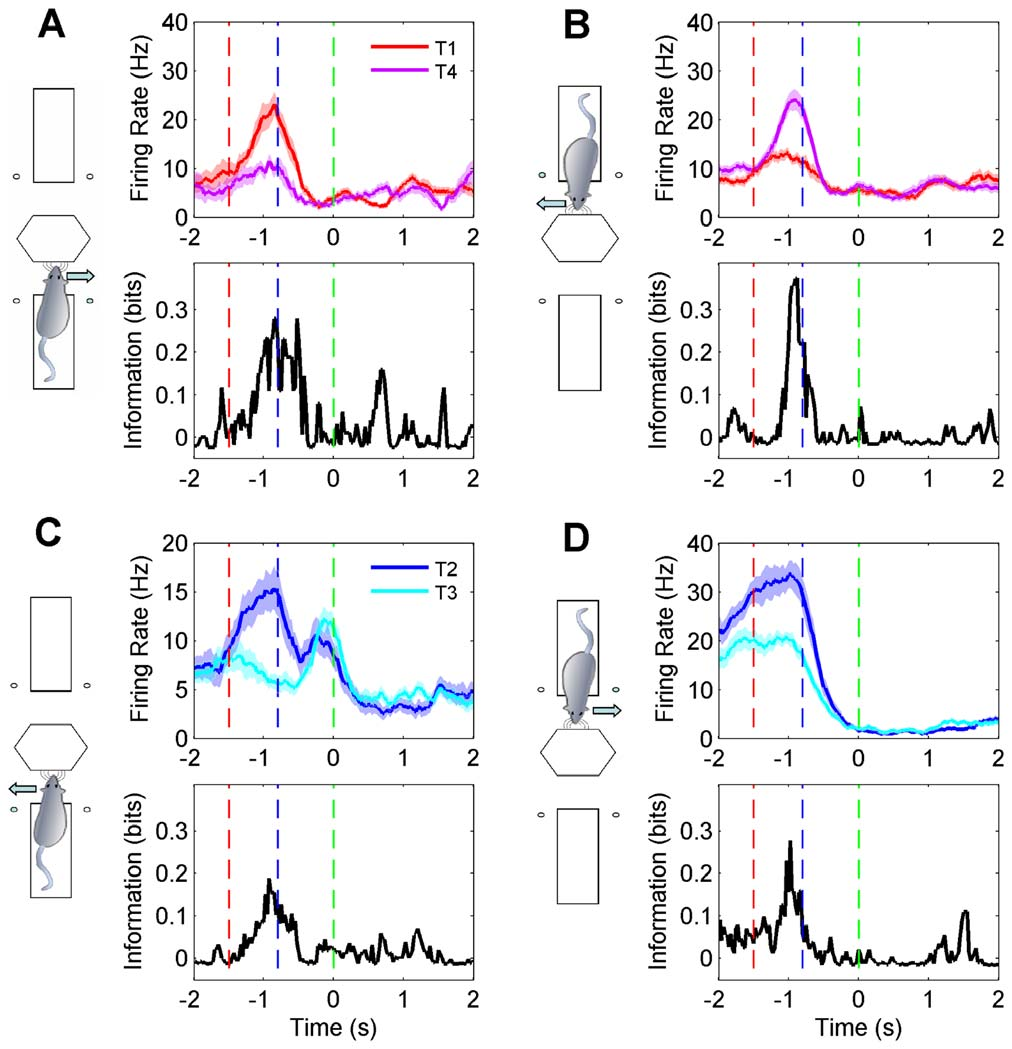
Texture signals in the two-platform task. For each of four neurons (A–D), responses are shown for the only pair of stimuli for which the value of texture information was statistically significant (p<0.05). The icon to the left of each plot shows the platform occupied by the rat and the action (arrows: turn left or turn right) associated with the two textures. In each panel, the upper plot shows time-varying firing rate related to the two textures. Shaded area corresponds to standard error of the mean. The lower plot shows the temporal profile of information about texture identity carried by neuronal firing rate. Red, blue, and green dashed lines indicate the estimated start of time windows during which rats engaged in touch, turn and reward collection.

In the one-platform task (rats 1–4), 15 out of 217 sampled neurons (7 percent) carried a significant texture signal (median value of peak information among informative signals: 0.16 bits, interquartile range: 0.12–0.19 bits). Texture information appeared when the animal began to touch the texture and in some neurons persisted or reappeared later in the trial, for example at reward onset. In the two-platform task (rats 5–6), 170 out of 679 sampled neurons (25 percent) carried a significant texture signal (median value of peak information among informative signals: 0.17 bits, interquartile range: 0.13–0.23 bits), considering both platforms together. On a given platform, over 13 percent of the sampled neurons encoded texture identity. Figure 3 shows four examples.

The texture signal was associated with a significant modulation of firing rate. Among putative pyramidal neurons that carried significant quantities of texture information, in the 400-ms window aligned with the peak texture signal the mean difference in firing rates evoked by the two encoded textures was 2.6 spikes per second, compared to a mean whole-session average firing rate of 2.5 spikes per second. Among putative interneurons, the same measure yielded a firing rate difference 4.1 spikes per second, compared to a mean whole-session average firing rate of 9.7 spikes per second.

### Neuronal Representation of Reward Location

During learning, dissimilar stimuli can become associated with the same action. This was the case in our experiment where, for example, in rats 1–2 disparate textures T1 and T3 both signified the presence of the reward to the right. In the preceding section, we showed that the firing of some neurons distinguished between different textures even when those stimuli were associated with the same reward location. Now we show that neurons also fired differently according to reward location (or, equivalently, to features bound to reward location such as the perceptual category of the paired stimuli, or the body turn towards the reward location).

To quantify neuronal coding, we labeled each trial by reward location, left or right. We then measured the information carried by neuronal firing rate in a 400 ms window that was advanced in 25 ms steps (the statistical criteria used to judge the significance of reward location information were similar to those used for texture information; see Materials and Methods).

**Figure 4:**
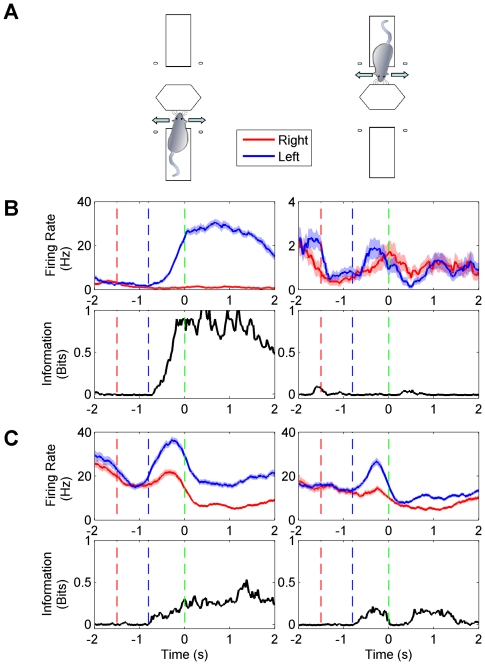
Representation of reward location. (A) The icon on the left indicates a platform A position: corresponding neuronal data are in the left panels of (B) and (C). The icon on the right indicates a platform B position: corresponding neuronal data are in the right panels of (B) and (C). (B) Neuron representing reward location on platform A (left) but not on platform B (right). Upper plots – time-varying firing rates aligned on the moment of reward delivery. Lower plots – temporal profiles of reward location information. In all response profiles, shaded area corresponds to standard error of the mean. Red, blue, and green dashed lines indicate the estimated start of time windows during which rats engaged in touch, turn and reward collection. (C) Neuron representing reward location on both platforms A and B, but with different strength and firing rates. The neurons illustrated in (B) and (C) did not encode texture.

In the one-platform task, 167 of 217 neurons (77 percent) carried a significant reward location signal. The median value of peak information among informative neurons was 0.22 bits, with an interquartile range of 0.12–0.42 bits. In the two-platform task, 402 out of 679 neurons (59 percent) carried a significant reward location signal. 241 neurons represented reward location on only one platform (Figure 4A) and 161 represented reward location on both platforms (Figure 4B). The median value of peak information among informative signals was 0.18 bits, with an interquartile range of 0.12–0.31 bits.

The firing of one neuron on trials in which the rat turned to the incorrect reward spout is given in Figure S3.

On average, reward location information appeared just after texture information and may in some cases reflect the animal passing into the neuron's place field. In many cases, reward location information persisted or reappeared later in the trial, for example at the moment when the water reward was released. In some neurons (see Table 1) reward location information appeared early in the trial, during the touch phase, and might reflect a prospective place code[15], [16].

**Table 1:** Numbers of neurons that carry significant amount of information about the texture (T) and reward location (RL) in all possible combinations separately for putative interneurons and pyramidal cells during each phase of the task together with the 99,9 percent confidence intervals (in brackets) expected from independent representations of texture and reward location.

	RL-T-	RL+ T-	RL-T+	RL+ T+
**Touch (Pyramidal Cells)**	825 (819∶831)	51 (45∶57)	85 (79∶91)	6 (0∶12)
**Touch (Interneurons)**	690 (684∶693)	43 (40∶49)	86 (83∶92)	6 (0∶9)
**Turn (Pyramidal Cells)**	642 (630∶653)	236 (225∶248)	65 (54∶77)	24 (12∶35)
**Turn (Interneurons)**	528 (520∶543)	205 (190∶213)	73 (58∶81)	19 (11∶34)
**Reward (Pyramidal Cells)**	585 (560∶591)	286 (280∶311)	56 (50∶81)	40 (15∶46)
**Reward (Interneurons)**	531 (512∶537)	210 (204∶229)	55 (49∶74)	29 (10∶35)

Note that in no case the real number of neurons lay outside the confidence interval range.

### Time Course of Texture and Reward Location Signals: Relation to Behavior and Hippocampal State

**Figure 5:**
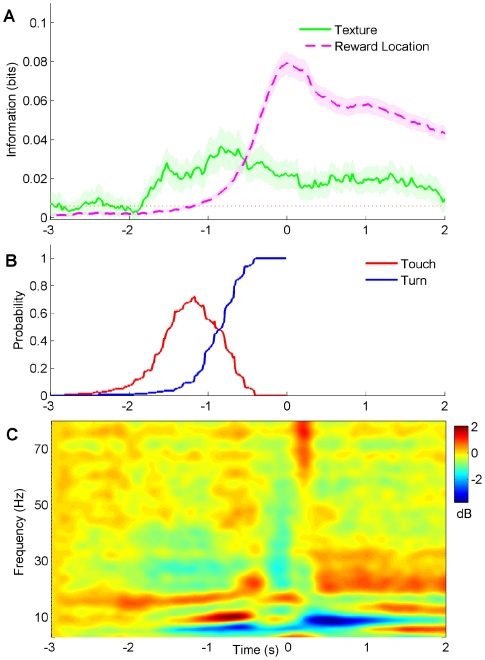
Relation between texture and reward signals, behavior, and local field potential oscillations. (A) Average temporal profile of texture and reward location information among all neurons with statistically significant signals. Horizontal red dotted line denotes average quantity of texture information in the interval 2–3 seconds prior to texture contact. Shaded area is standard error of the mean. (B) Probability distribution of touch and turn epochs in behavior, aligned to reward onset, reproduced from Figure 2C. (C) Event related spectral perturbation of local field potential. Color bar represents change in power in dB relative to the interval 2–3 seconds prior to texture contact.

The temporal profile of texture and reward location signals, when aligned with measures of hippocampal state, can provide insights into the mechanisms at work. From the neuronal activity recorded during both the one-platform and two-platform tasks, we averaged together the 210 statistically significant temporal profiles of texture information, as well as the 730 statistically significant temporal profiles of reward location information. (In the two-platform task, a single neuron could contribute more than one profile to the average.) Texture information (Figure 5A) increased above baseline when touch probability reached about 20 percent (compare to behavior time course in Figure 5B), about 1.7–1.8 seconds before reward onset. The average texture information in the population of neurons then rose together with touch probability. The largest signal occurred about 800 ms before reward onset – at that point, the rats' behavior switched from touch to turn in the typical trial.

Reward location information departed from the baseline level 600–700 ms after the onset of texture information (Figure 5A). This was 1.0–1.1 seconds before reward onset. As the probability of the rat turning towards the water spout rose, reward location information rose in parallel. The peak in reward location information occurred during the 100 ms interval centered on reward onset, about 800–900 ms after the peak in texture information. For both texture and reward location signals, there was a slight increase in strength 500–800 ms after reward onset.

A crucial finding is that the texture signal did not approach zero even after contact with the texture ended: it was conserved as the animal turned towards the reward location and even as the reward was delivered. How did the hippocampal network maintain a memory trace for the contacted texture once the sensory system no longer provided such input? Individual neurons carried texture information only in short bursts, with an average total duration between 400 and 500 ms (Figure 3). This suggests that information was maintained by network dynamics rather than by continuous single-neuron memory traces (also see Figure S5).

The behavior was accompanied by transitions in brain state, revealed by the spectrogram of the CA1 local field potential (Figure 5C). During approach to and contact with the texture, there was a significant increase in power in the theta and beta range (7–12 Hz and 15–20 Hz, respectively) relative to the intertrial period (p<0.0005, permutation test, Bonferroni-corrected). The increase was probably related to increased motor activity[17]. During reward collection, there was a sharp drop in theta power (p<0.0001), and an increase in the beta and lower gamma range (20–35 Hz; p<0.005). Theta power remained very low throughout the period of reward collection (Figure S6). We observed very few ripple events during performance of the task (Figure S7).

### What Is the Nature of the Texture Representation in CA1?

In the hippocampus, responses to visual, olfactory, auditory stimuli and objects are modulated by the location of the rat[18]–[21]. From this, our expectation was that the representation of texture by individual neurons would vary according to the context in which the stimulus was encountered.

Texture sensation begins when the animal palpates a surface to generate a texture-specific pattern of whisker motion[1], [2], [22]. Whisker motion is converted to spike trains by receptor neurons and transmitted along the sensory pathway to the primary sensory cortex. In sensory cortex, texture coarseness is positively correlated with neuronal firing rate[1], [3], [4]. We asked whether CA1 neurons encode textures as physical features or as context-embedded events. If coding were related to the physical features of textures, two findings would emerge. First, the neuronal population on the whole would show a systematic input/output relationship, giving a stronger response for some stimuli than for others. Second, individual neurons would fire in a consistent manner for a given stimulus, independently of the location of the animal. We tested the first prediction by identifying the texture that evoked the peak firing rate for all 147 neurons that discriminated between a single texture pair in the two platform task. The number of neurons that preferred each of the textures was: T1, 45 neurons; T2, 37 neurons; T3, 22 neurons; T4, 43 neurons. This distribution indicates no systematic relationship between stimuli and peak firing rate.

We tested the second prediction by determining whether texture signals “followed” single neurons as stable attributes. The most direct evidence against this notion is that in the two-platform task, 147 neurons (those referred to above) encoded texture on just one platform: they failed to distinguish between the same textures when the rat was positioned on the opposite platform. Moreover, among 21 neurons that encoded a single texture pair on each platform, only 6 demonstrated consistent “texture tuning”; the remaining 15 either discriminated between the same texture pair on both platforms but switched preference within the pair, or else discriminated between different texture pairs on the two platforms. In conclusion, texture in hippocampus was represented in conjunction with the context, with no relationship to the physical features of the stimuli. Moreover, texture representations “remapped” when the animal performed the task in different locations, as has previously been demonstrated for the representation of space[23] and objects[21]. These observations differ from the coding scheme uncovered in human temporal lobe neurons[24], where neurons can represent objects across different contexts.

### Relationships among the Signals Present in CA1

**Figure 6:**
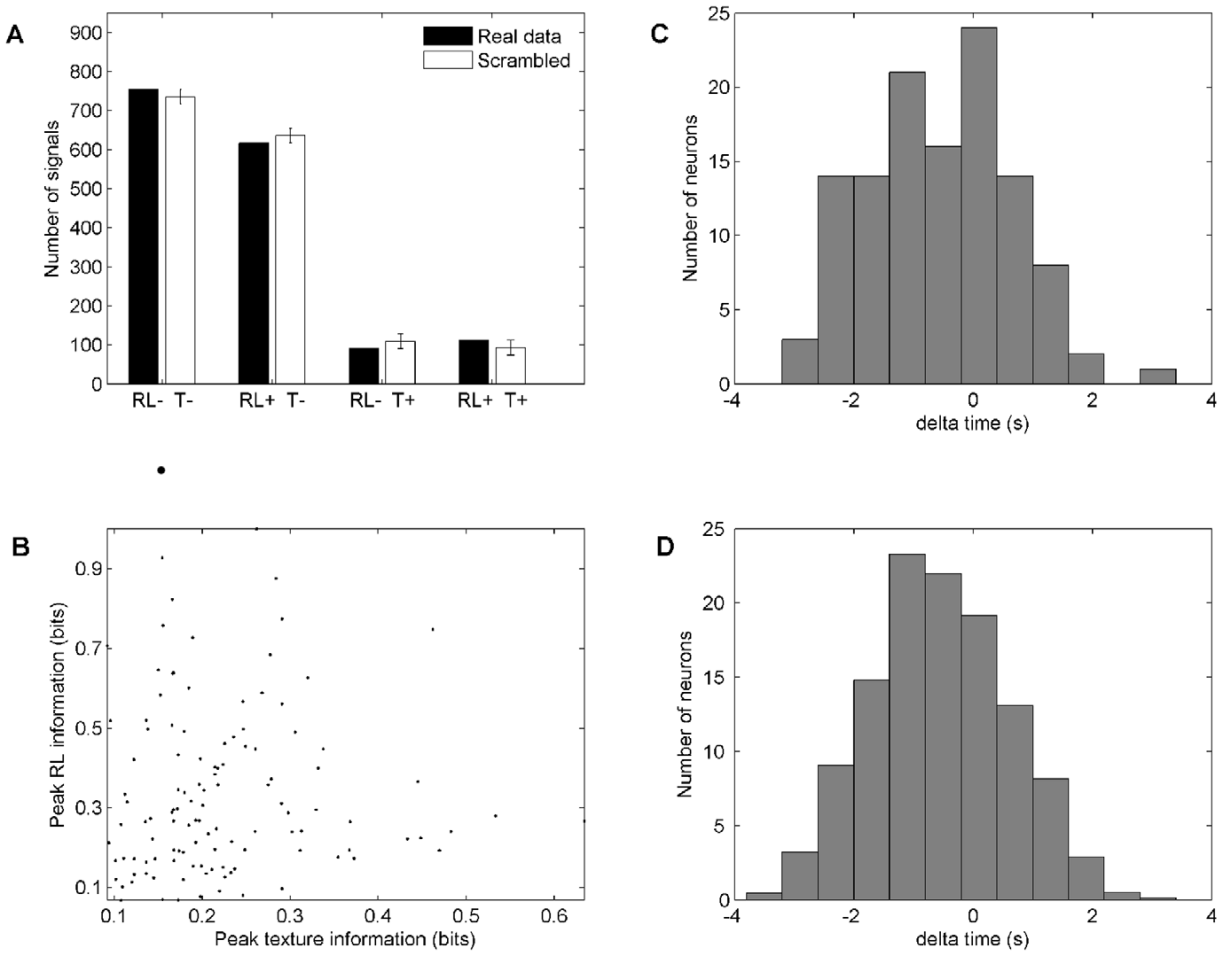
Relationship between texture signals and reward location signals. (A) Test to determine whether the presence of one type of signal predicted the presence of the other type. Black bars indicate the numbers of signals about neither texture information nor reward location information (RL+, T-), just one of the two (RL+, T- or RL+, T-), or both (T+, RL+). Data from the two platforms were treated separately. The white bars indicate the count of signals that would be expected to carry given combinations of signals if texture and reward location were encoded independently, with 95 percent confidence intervals included. The simulated and observed distributions are identical. (B) Test to determine whether the strength of one type of signal predicted the strength of the other type. The analysis was applied to those neurons that carried significant quantities of both texture and reward location information (T+, RL+) on the same platform. There was no significant correlation between the two (Spearman Rho  = 0.1, p  = 0.2). (C-D) Test to determine whether the time of occurrence of one type of signal predicted the time of occurrence of the other type. The upper panel shows the time of peak texture information - time of peak reward location information among neurons that carried both signals (T+, RL+) on the same platform. The lower panel shows the same analysis, but time differences were measured between rather than within neurons, in 1,000 random combinations. The two distributions are equivalent, suggesting that within individual neurons there was no causal relationship between the appearance of the reward location signal and the appearance of the texture signal.

Were texture and reward location represented independently or did neurons have an affinity to one or the other modality? To answer this, we counted the numbers of neurons that carried each of the four possible signal combinations: texture and reward location, only texture (Figure S8), only reward location, and neither. The presence of a signal was based upon the average information carried across all phases of the task. The black bars in Figure 6a give the counts of such neurons. The white bars give the counts of neurons that would be expected to carry the corresponding signals according to a permutation test that assumes independence of texture and reward location signals. The true distribution perfectly matches the simulated one. The equivalence between the two distributions signifies that the presence of one signal does not influence the likelihood of carrying the other signal.

We also spliced the behaviour into three discrete phases (touch: −1500 to −800 ms; turn: −800 to 0 ms; reward: 0 to 1000 ms) and again counted the numbers of neurons that carried each of the four possible signal combinations (Table 1). The data set was divided into pyramidal cells and interneurons. In both types of neuron, texture and reward location signals were independent in *each single phase* of the behaviour. The importance of this observation is that the population simultaneously, not just sequentially, carried multiple signals independently.

After finding that the presence of texture and reward signals was independent, we investigated their relationship in magnitude and in time. Among the neurons that carried information about both texture and reward location on the same platform, we asked whether the peak strength of one signal predicted the peak strength of the other. We didn't find any correlation between the two values (Spearman Rho = 0.1; p = 0.2) (Figure 6B). Next, we measured the temporal delay between the two peaks in information (Figure 6C, upper panel). Within single neurons the texture peak on average occurred earlier than reward location peak, as expected from the overall time courses of the two signals (Figure 5A). When the peak times of texture and reward location signals were compared across randomly selected pairs of neurons (Figure 6C, lower panel), the mean and spread of the distribution is unchanged. This means that the two signals were no more correlated in time, in single neurons, than could be expected by chance. Both the magnitude and the timing results suggest that, even among the neurons that carried both texture and reward location signals, there was no substantial relationship between them. For the two-platform experiment, we could additionally ask to what extent the presence of a texture signal at one location in the arena influenced the likelihood of texture signals at other locations. The result is that texture signals, in different locations, were independent events with no influence on each other (Figure S9).

**Figure 7:**
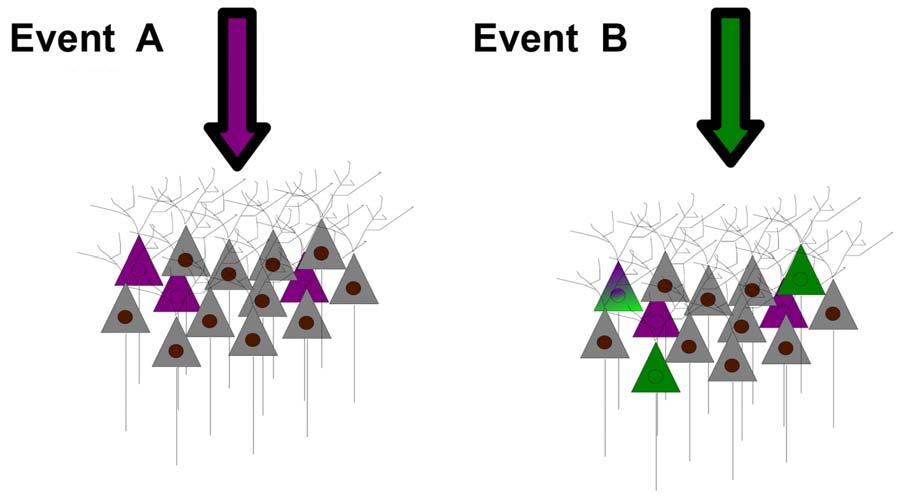
Independence of populations recruited to encode different events. When the hippocampus receives input specifying the occurrence of event A (magenta arrow), one subset of neurons is recruited to encode that event (magenta-tinted neurons). On the occurrence of event B (green arrow) another subset of neurons is recruited (green-tinted neurons). Because separate events are represented in an independent, partially overlapping manner, some neurons will be recruited to represent neither event, some neurons just one of the two events, and some neurons both events (note neuron with mixed color). A and B may be two events of the same modality in different contexts, such as the encoding of texture on two different platforms. Alternatively, A and B may be two events of different modality that occur in the same context, perhaps even occurring at the same time, such as the encoding of texture and reward location on the same platform.

These findings all lead to the idea that tactile stimuli and the reward location associated with them are represented independently in the CA1 region of hippocampus. The results are best explained by a model where the recruitment of neurons for the representation of one event occurs independently of the recruitment of neurons for the representation of a second event, whether the two events are sequential or simultaneous (Figure 7).

## Discussion

### Representation of Touch in the Hippocampus

This work provides a characterization of tactile encoding in rat hippocampus. Unlike the texture representation in barrel cortex, the representation in hippocampus does not reflect the physical properties of the stimulus; there was no tendency for coarser textures to evoke a greater firing rate. The texture representation was modulated by the animal's location – neurons that discriminated between a texture pair in one position frequently failed to discriminate between the same texture pair in a different position. In conclusion, neurons did not encode textured stimuli as physical objects along the dimension of coarseness, but as meaningful events in conjunction with the location in which they appeared.

Stimulus-related firing has been previously described in hippocampus in the olfactory[25]–[27], auditory[20], and visual[28] modalities as well as in spontaneous object recognition[21]. In one publication[29] neurons in awake, behaving rats also were interpreted as encoding tactile signals, but the two touch inputs in question (contact with widely versus narrowly spaced lateral walls) were associated two different reward locations. Neurons considered to be tactile might have fired differently only according to the behaviour (turn right versus turn left) that the rats expressed when they contacted tactile stimuli. In contrast, in our work signals were taken to be tactile only when neurons fired differently for two different textures associated with the same reward location.

### Persistence of texture signals: Bridging the temporal gap?

Separating tactile from reward location signals allowed us to track both signals over time. Neuronal information about the palpated texture did not disappear when the whiskers broke off contact with discriminandum as occurs in early stages of sensory processing, but persisted and even showed a slight increase in strength one-half second after reward onset (Figure 5). The average time courses in Fig. 5A were generated from single-neuron profiles of many different shapes. These included single peaks (early, late, or intermediate) and multiple peaks. No neuron maintained information continuously throughout the whole trial. The transient nature of the information in single neurons, in contrast to the continuous signal seen in the population average, brings to mind an intriguing model of short term information storage: the signals may be “handed off” locally in CA1 from neuron to neuron from the time of first stimulus contact until some time after reward consumption (Figure S5). On the other hand, non-local mechanisms could account for the memory trace; signals might be stored outside the hippocampus, for example in prefrontal cortex[30]–[33] or in entorhinal cortex[34], and updated constantly via direct connection. In alternative, increased low gamma activity (20–35 Hz) during reward collection (Figure 5C) might reflect increased functional connectivity between CA3 and CA1 fields of hippocampus[35]; it is possible that texture signals were preserved in the recurrent network in CA3 and were retrieved when the animal received the reward. A continuous representation of texture, in the absence of the stimulus itself, might rely on mechanisms similar to those underlying the internally generated representation of space when rats run on a wheel during the delay period of a continuous alternation task[36].

What might be the function of the persistence and recurrence of texture information? We suggest it reflects a mechanism whereby an early input is “stretched” in time to be made contiguous with a later input, ultimately allowing the rules of synaptic plasticity to bind the events together[37]. In our experiments, texture, reward location, and reward acquisition could become associated so that the presence of one cue draws up the recall of the others.

### Allocation of independent subsets of neurons into the representation of events: Snapshot memory?

Our findings contain elements consistent with the theoretical frameworks proposed to explain the specificity of hippocampal firing for places and for objects. According to relational theory[11], [25], [38], hippocampal neurons represent events in their context, for example an object encountered in relation to the location of that object. The linkages between objects and context could form a neuronal basis of episodic memory[38]–[41]. Cognitive map theory based upon the hallmark “place cells”[42], postulates that hippocampus is crucial in the formation of a navigational map of the environment. In an extension of the theory, neuronal activity within its place field is modulated by the presence of objects or altered cues in the arena[18], [23], [43]. In our experiments, the conjunctions between texture and reward location signals are consistent with relational theory, while the strong influence of the animal's location on neuronal firing is consistent with cognitive map theory. A new insight is the concept of independence – the likelihood of the conjunctions of features (i.e. texture and reward location) was equal to the product of the prior likelihoods of representing either feature separately. From the point of view of the population, in each context the set of neurons engaged to represent a given feature was independent of the set of neurons engaged to represent a different feature; moreover, the set of neurons engaged to represent a given feature in one context was independent of the set of neurons engaged to represent the same feature in a different context (Figures 6, and Figure S9). The most plausible interpretation, as illustrated in Figure 7, corresponds to snapshot-like representations of objects and the context which surrounds them, an interpretation that resembles some notions of episodic memory[39].

The coding properties of neurons did not “follow” them across contexts. If a neuron's intrinsic properties do not determine whether it will be engaged to participate in the representation of a given event, what process selects neurons? One possible mechanism underlying this phenomenon is that the responding population is initially selected based on random synaptic connectivity, which gives certain neurons a larger initial input[44]. Alternatively it may depend only on the transient state of the neuron (random fluctuations of the membrane potential, levels of calcium, etc.) at the initial occurrence of the sensory event. A scheme of this sort for memory storage was proposed by David Marr[45] as a simple memory system, which he identified with archicortex in general and hippocampus in particular.

## Materials and Methods

### Ethics Statement

All experiments were conducted in accordance with National Institutes of Health, international, and institutional standards for the care and use of animals in research and were approved by the Bioethics Committee of the International School for Advanced Studies (permit n.5575-III/14) and were supervised by a consulting veterinarian.

### Subjects

Six Wistar rats weighing about 350 g were housed individually and maintained on a 14/10 light/dark cycle. Water was given during training as a reward and was also available ad lib for 1 h after training.

### Apparatus

The arena was situated in a Faraday room and was illuminated by light-emitting diodes discharging at infrared wavelength (880 nm). There were four acrylic glass discriminanda (Figure S10). All textures had the same size, shape and odor. Potential olfactory cues were removed by washing the textures at least once every session. Several different exemplars of the same textures were used to make sure that rats did not use specific cues attached to one particular object. In addition, a previous study demonstrated that during incorrect trials whisker-mediated neuronal signals were altered[46] which allowed us to conclude that this behavior is whisker dependent.

### Texture discrimination on one platform

First, the rats learned to discriminate between two textures associated with opposite reward locations. For a given texture, the rat was given a water reward (0.1 ml) only at one of the drinking spouts; at the incorrect spout it received no water. When it reached stable performance (on average 2–3 weeks), one of the textures was substituted with a new discriminandum and training continued until the performance became stable again (2–4 days). As a next step the three textures were presented together: now, two textures were associated with one reward location and the third was associated with the opposite location. The texture-reward location associations were fixed across all sessions (Table S1). This training on average took 6 weeks.

### Texture discrimination on two platforms

Rats 5–6 were trained on this task. At the outset, they were trained on platform A (rat 5) or B (rat 6) until they learned the correct action for all four textures; this took 7–8 weeks. Next, they were trained on the opposite platform; the association between each texture and its required action was held constant in self-centered coordinates. Before and after the training session, the room was illuminated by visible light so that rats could be aware of the two distinct platforms and the spatial relation between them. The platforms differed not only in the position within the room but also by their floor texture.

### Surgery and recording

Rats were anaesthetized with a mixture of Zoletil (30 mg per kg) and Xylazine (5 mg per kg) delivered *i.p*. A craniotomy was then made above left dorsal hippocampus, centered 3.0 mm posterior to bregma and 2.5 mm lateral to the midline. A microdrive loaded with 6 (rats 1–4) or 12 (rats 5–6) tetrodes (25 micron wire Pl/Ir wire) was implanted over the craniotomy. Tetrodes were moved individually until they reached CA1. A neuron could be recorded across multiple, contiguous sessions if its spike waveform and functional properties were perfectly stable over time. In those cases, the data across sessions were merged and counted as just one data point.

### Data analysis

Our main hypothesis was that the firing of hippocampal neurons represented various components of the touch-guided behavior – specifically, the stimulus identity and the explicit actions (places) associated with that stimulus. Therefore, we needed to estimate the quantity and statistical significance of the signal carried by the firing rate modulation of individual neurons on single-trials. For this purpose, we employed Shannon's Mutual Information[47], hereafter referred to simply as “information”.

### Information Measures

The information that any signal X conveys about a second variable Y can be described as:
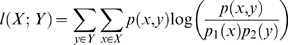
(1)


In our case, *X* refers to the parameter that defines a single trial (texture or reward location) and *Y* refers to neuronal spike count in a specific temporal window. This quantifies the reduction of uncertainty about the trial parameter (*X*) gained by a single-trial observation of the spike count (*Y*). The probabilities in the above formulas are not known *a priori* and must be estimated empirically from a limited number, *N*, of trials. Limited sampling of response probabilities can lead to an upward bias in the estimate of mutual information[48], [49]. An approximate expression for the bias has been formulated[48] and can be subtracted from direct information estimates (Eq. 1), provided that *N* is at least two to four times greater than the number of different values that *X* can assume[50]. In our data, *N* was at least 12 and *X* could assume 2 values.

### Texture and reward location information carried by the temporal profile of response

We measured the information carried by spike counts in a 400 ms window sliding in steps of 25 ms along the whole duration of the trial, from 2 seconds before the animal received water reward to 2 seconds after. The 400 ms integration window was selected as the one providing the most significant average information values for both reward location and texture information. The analysis provided a temporal profile of information whose overall strength could be verified by the statistical test below.

### Statistical tests for the identification of a texture signal

Texture information quantified how reliably a neuron's firing rate could distinguish between the two stimuli associated with the same reward location. In every 400 ms window, the set of firing rates across trials provided one value of information, and shifting the window in 25 ms steps gave 145 sequential information values. To measure the significance of a neuron's signal, we used a procedure based on comparison of the observed quantity of information, averaged across 4 seconds, to a distribution of simulated values. Each simulated value was obtained by scrambling across trials the texture labels for the texture pair associated with the same reward location, computing the temporal profile of information, and then finding its average value. This is an estimate of the average information expected if texture had no systematic influence on neuronal firing. The true value of average information was judged to be significant if it passed a threshold of p<0.05.

### Statistical tests for the identification of a reward location signal

Reward location information quantified how reliably a neuron's firing rate could distinguish between the two behaviors expressed on a single platform – turn left versus turn right. To measure the significance of a neuron's signal, we used a two-step procedure. The first step was similar to the texture information measure except that trials were pooled based on the side of the reward, so that on every trial the response was labeled left or right, with texture identity dropped. The true value of average information was judged to be significant if it passed a threshold of p<0.05. A second step was added to guard against potentially spurious reward location signals. Suppose that a neuron gave a large response to T1 (right) but no response at all to T2 (left), T3 (left), and T4 (right). The strong T1 response would make the neuron appear to fire more for the right reward location. This might lead to a significant value of reward location information, which should be considered false because presentation of the second texture of that pair (T4) evoked no corresponding reward location signal. To exclude any such cases, the reward location information procedure was repeated with all permutations of texture pairs. In the example above, simulated reward location groupings would be T1–T2 versus T3–T4, and T1–T3 versus T2–T4. The neuron was considered significant if it carried significant signal (p<0.05) for all of the four conditions.

To determine whether neurons carried significant texture or reward location signals during specific phases of the task, we divided the trials into three discrete phases (Touch: −1500 to −800 ms; Turn: −800 to 0 ms; Reward: 0 to 1000 ms) and followed the procedures given above for restricted time windows.

### Independence between texture signals and reward location signals

We used three tests to determine whether neurons encoded sensory information in a manner that was correlated with, or independent of, spatial information. The first measured whether the presence or absence of one type of signal predicted the presence or absence of the other type. The second measured whether the magnitude of one type of signal predicted the magnitude of the other type. The third measured whether the time of occurrence of one signal predicted the time of occurrence of the other signal.

The first test consisted of several steps: (i) We constructed a matrix in which each row referred to one neuron. In the first column, an entry of 1 or 0 indicated that a given neuron did, or did not, carry a significant texture signal for any of the 2 possible reward locations on a given platform. In the second column, an entry of 1 or 0 indicated that a given neuron did, or did not, carry a significant reward location signal on the same platform. Data from the two platforms were treated separately. There were 1575 rows (679 neurons, 2 platforms from the 2 platform task and 217 neurons from the one platform task) and 2 columns (texture and reward location). (ii) We counted the number of neurons that carried neither texture nor reward location signals [RL-, T-], the number that carried one but not the other [RL+, T-] or [RL-, T+], and the number that carried both texture and reward location signals [RL+, T+]. (iii) To interpret the population distribution, we scrambled the entries within each column of the matrix, thus making a given neuron's texture and reward location signals independent of each other, without altering the total number of texture and reward location signals in the neuronal population. (iv) After the shuffle, we again counted the number of neurons that carried each of the possible combinations of texture and reward location signals. Steps (iii) and (iv) were repeated 1,000 times and the mean number of neurons carrying a specified combination of signals was calculated, along with 95 percent confidence intervals.

The second and third tests were applied only to those neurons that carried a significant quantity of both types of signal, judged by the average value across the entire trial. To look for a potential correlation in signal strength, we measured the texture and the reward location information in a sliding 400 ms window and registered the peak values of each signal, regardless of when either signal occurred. To look for a potential correlation in timing, we calculated the time difference, delta-t, of occurrence of the peaks of the two signals and plotted all values (Figure 6c, upper panel). Then we calculated the time difference in the peak of texture and reward location signals, but taking each signal from a different neuron. The shuffling procedure was repeated 1,000 times and all values were plotted (Figure 6c, lower panel).

### Histology

The animals were anesthetized and transcardially perfused with 10 percent formalin. Brains were sectioned in the coronal plane and stained with cresyl violet. Electrode tracks were localized on the serial sections.

## Supporting Information

Table S1 **Experimental conditions.**
(DOC)

Video S1 (AVI)

Figure S1 **Spike sorting and histology.** (A) Waveforms of seven single units isolated from one tetrode. This recording is typical of those in which more than 5 neurons were isolated. Vertical scale bars represent 150 microvolts. (B) Scatter plot of waveform energy from two channels of the tetrode demonstrating separation of units; grey dots are events unaccounted for by any cluster. (C) Histological section. Red dashed oval indicates electrode track within CA1 subfield of hippocampus.
(TIF)

Figure S2 **Separation between pyramidal cells and interneuron's.** Three criteria were used to separate pyramidal cells: mean firing rate[51], [52], spike duration[53], and the autocorrelation function[54]. Firing rate was measured over the whole session. Spike duration was initially measured from peak-to-valley[3] and at 25% of maximum spike amplitude[54]; the former measure proved to be more reliable and was used in all sessions. The autocorrelation-derived index assessed the ratio of the difference between the number of spikes that occurred in a 2–5 ms post-spike window versus a 20–80 ms post-spike divided by the sum of the two. Spike duration and the autocorrelation-derived index yielded bimodal distributions. Based on firing rate, spike duration, and the autocorrelation-derived index, the complete set of neurons were clustered into 2 classes using a K-Means algorithm. Spike duration was longer in pyramidal cells (0.44±0.004 ms) compared to interneurons (0.25±0.0062 ms), consistent with[53] (interneurons' spike duration <0.3 ms, pyramidal cells' spike duration >0.3 ms)3. Putative pyramidal cells and putative interneurons had distinct autocorrelation profiles. Interneurons had significantly higher firing rate than pyramidal cells (13.06±0.68, and 2.63±0.16 spikes/s, respectively, Wilcoxon rank sum test p<0.000001). The firing rate of our pyramidal cells appears to be slightly higher than previously reported (e.g. 1.4±0.16 in[54]). We assume it is due to the fact that our apparatus was small and the majority of the recording was done during the task, so the probability of the discharge of a place cell would be higher compared to the standard task in which a rat runs in a larger maze.
(TIF)

Figure S3 **Firing of reward location neurons during incorrect trials.** In our experiments, the number of error trials was low. For this reason, we were not able to obtain clear or stable statistical results on error trials. Nevertheless, in a few cases, there was a sufficient number of error trials, and with the same temporal rhythm, to allow spike times to be reliably aligned to behavior. For the neuron illustrated here, when the rat turned to the Left reward location in error (C, left column) the response profile closely resembled that present when the rat turned Left reward location on correct trials (B, left column). This indicates that the neuron's firing was correlated with the behavior of the rat. It does not specify, of course, which aspect of the behavior the neuron tracked – the perceived stimulus category, motor action, or the place. (A) Icon indicating the position of the animal in the behavioral setup. (B) Firing during correct trials, (C) firing during incorrect trials.
(TIF)

Figure S4 **Texture neurons meta-analysis.** To estimate the probability of finding the number of neurons with the significance threshold of p<0.05 we have performed a meta-analysis of the data. The distribution in blue represents the number of neurons which is expected to surpass our threshold of significance (scrambled) in averaged across all four reward location and the red bar indicates the real number of texture neurons on average associated with any given reward location in the real data. The p value of 0.0000000002 (z-test) indicates that it was highly unlikely to find such number of neurons that carry significant amount of texture information by chance.
(TIF)

Figure S5 **Information about texture is distributed across neurons over time.** Information profiles of 4 simultaneously recorded neurons. Only traces of information associated with right turn on platform B are shown. Note that simultaneously recorded neurons carried information about texture at different points in time. Such a distribution of information was typical and led to Figure 5a when all traces were averaged.
(TIF)

Figure S6 **Reward-aligned local field potentials in a single trial.** (A) Raw local field potential voltage trace (black) and 5–12 Hz (theta) filtered voltage trace (red). Red, blue, and green vertical dashed lines indicate the estimated start of time windows during which rats engaged in touch, turn and reward collection. (B) 18–35 Hz (beta and low gamma) band pass-filtered voltage trace. (C) 120–240 Hz band pass-filtered local voltage trace.
(TIF)

Figure S7 **Ripple events.** (A) Raw (unfiltered) local field potential voltage trace aligned on the maximum of the ripple event. (B) 120–240 Hz band pass filtered local field potential of the same voltage trace. (C) Raster plot of 23 simultaneously recorded neurons aligned on the ripple event. Red – pyramidal cells, blue – interneurons. (D) Relationship between ripple events (red X) and trials. Each blue rectangle represents a 4-second trial centered on the moment of reward onset. Ripples occurred most commonly when the rat took an extended pause within the session. (E) Cumulative probability of observing a ripple event as a function of distance in time from the closest reward delivery event. (F) Probability of observing a ripple event from the closest in time reward delivery event. Plots (E) and (F) indicate that the occurrence of a ripple event during the behavioral task was extremely rare. No cells were found to be active only during sharp waves (defined according to[55]). The cell whose activity was most attributable to sharp waves fired just 7% of its spikes during the waves.
(TIF)

Figure S8 **Representation of texture without reward location.** Presence of a texture signal does not require the presence of a reward location signal. (A) The firing rates profiles associated with the animal's selection of the left and right reward locations were overlapping at all times on platform A. (B) Firing rate profiles associated with the left reward location on platform A are separated according to the texture present on each trial; now stimulus-specific activity can be discerned. (C) Temporal profile of information about reward location carried by neuronal firing rate; values were close to zero (black). In contrast, large quantities of texture information (blue) were present both during contact time and during reward collection.
(TIF)

Figure S9 **Independence of populations recruited to encode texture in different locations.** In the two-platform task, neurons were tested for the presence of texture information on four occasions (the left and right texture pairs on platform A, and the left and right texture pairs on platform B). We asked whether the presence or absence of information about texture within one pair influenced the probability that a neuron would carry information about texture within any other texture pairs. Black bars indicate the numbers of neurons that carried a given number of texture signals, from 0 to 4. Of all 170 “texture neurons”, the majority (147) distinguished between only one texture pair on only one platform (for example, those neurons illustrated in Figure 3). Just 24 of 170 carried signals for more than one texture pair. The white bars indicate the count of neurons that would be expected to carry a given number of texture signals if encoding of each texture pair were independent, with 95% confidence intervals included. The simulated and observed distributions are closely matched. We speculate that the independence of the tactile representations on the two platforms was an outcome of the rats' interpretation of the platforms as two independent contexts; they needed to be retrained on the second platform and showed consistently lower performance there (see **Touch-guided Behavior**). We suggest that the hippocampal representation of salient events underwent a reset each time the rat was moved between platforms.
(TIF)

Figure S10 **Tactile stimuli.** Photographs of rough tactile discrimanda, textures 2–4 (T2–T4). T1 was a smooth plate. Scale bar is 10 mm.
(TIF)
