# Supplementary material for: Hippocampal Representation of Touch-Guided Behavior in Rats: Persistent and Independent Traces of Stimulus and Reward Location
Source: PLoS One. 2011 Jan 28;6(1):e16462. doi: 10.1371/journal.pone.0016462 (PMC3030589; doi:10.1371/journal.pone.0016462)
Supplement: Table S1 — Experimental conditions. (DOC) [file pone.0016462.s001.doc]

**Table S1. Experimental conditions.**

| **Experiment** | **Textures: Right**  **reward location** | **Textures: Left reward location** | **Training and recording** | **Number of neurons** |
| --- | --- | --- | --- | --- |
| rat 1 | 1, 3 | 2 | Platform A | 77 |
| rat 2 | 1, 3 | 2 | Platform A | 57 |
| rat 3 | 1,2 | 3 | Platform A | 49 |
| rat 4 | 1, 3 | 2 | Platform A | 34 |
| rat 5 | 1, 4 | 2, 3 | Platforms A and B | 363 |
| rat 6 | 1, 4 | 2, 3 | Platforms A and B | 316 |
| **Total** |  |  |  | **896** |
