# Supplementary figures and images for: Hippocampal Representation of Touch-Guided Behavior in Rats: Persistent and Independent Traces of Stimulus and Reward Location
Source: PLoS One. 2011 Jan 28;6(1):e16462. doi: 10.1371/journal.pone.0016462 (PMC3030589; doi:10.1371/journal.pone.0016462)

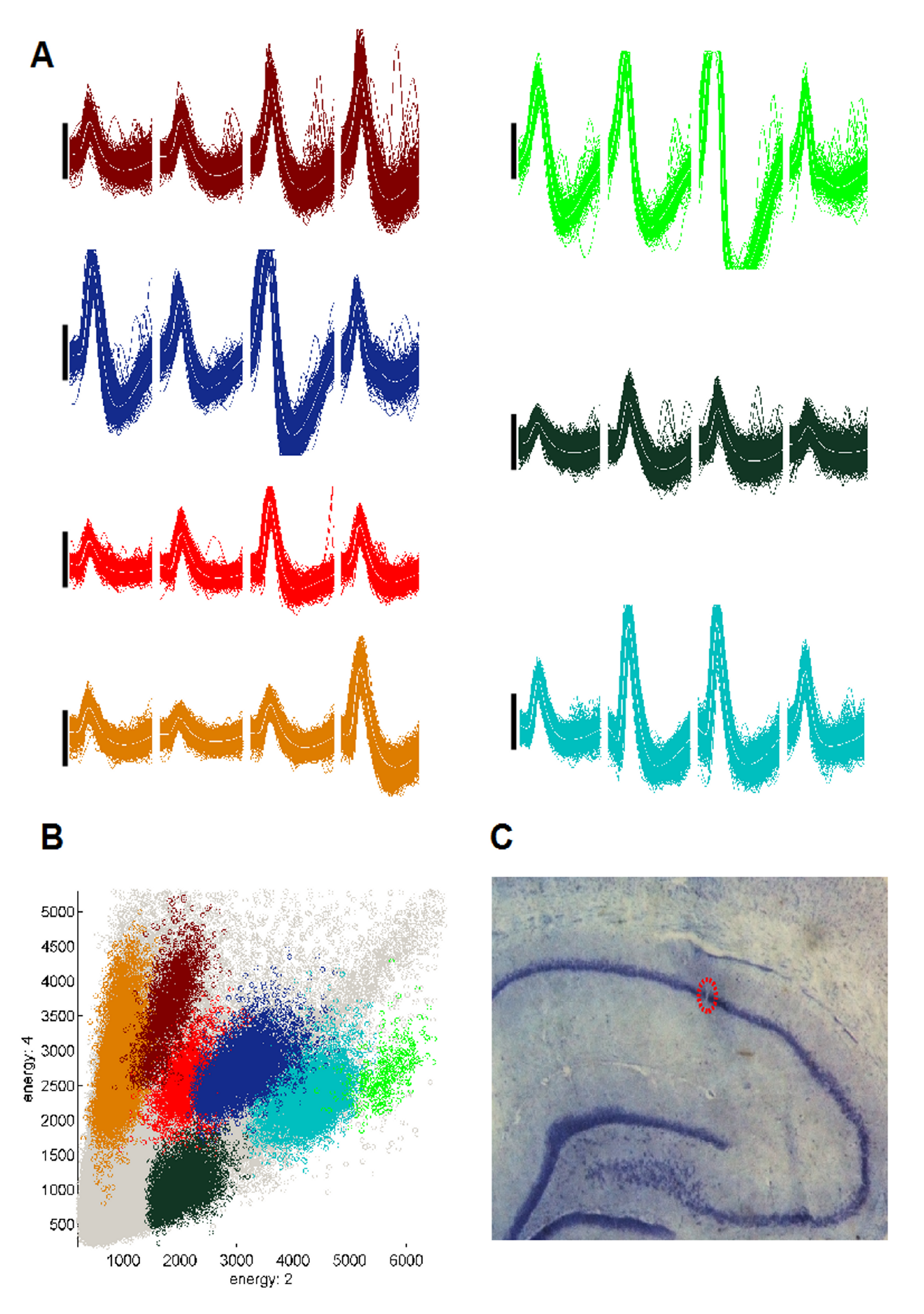

Supplement: Figure S1 — Spike sorting and histology. (A) Waveforms of seven single units isolated from one tetrode. This recording is typical of those in which more than 5 neurons were isolated. Vertical scale bars represent 150 microvolts. (B) Scatter plot of waveform energy from two channels of the tetrode demonstrating separation of units; grey dots are events unaccounted for by any cluster. (C) Histological section. Red dashed oval indicates electrode track within CA1 subfield of hippocampus. (TIF) [file pone.0016462.s003.tif]

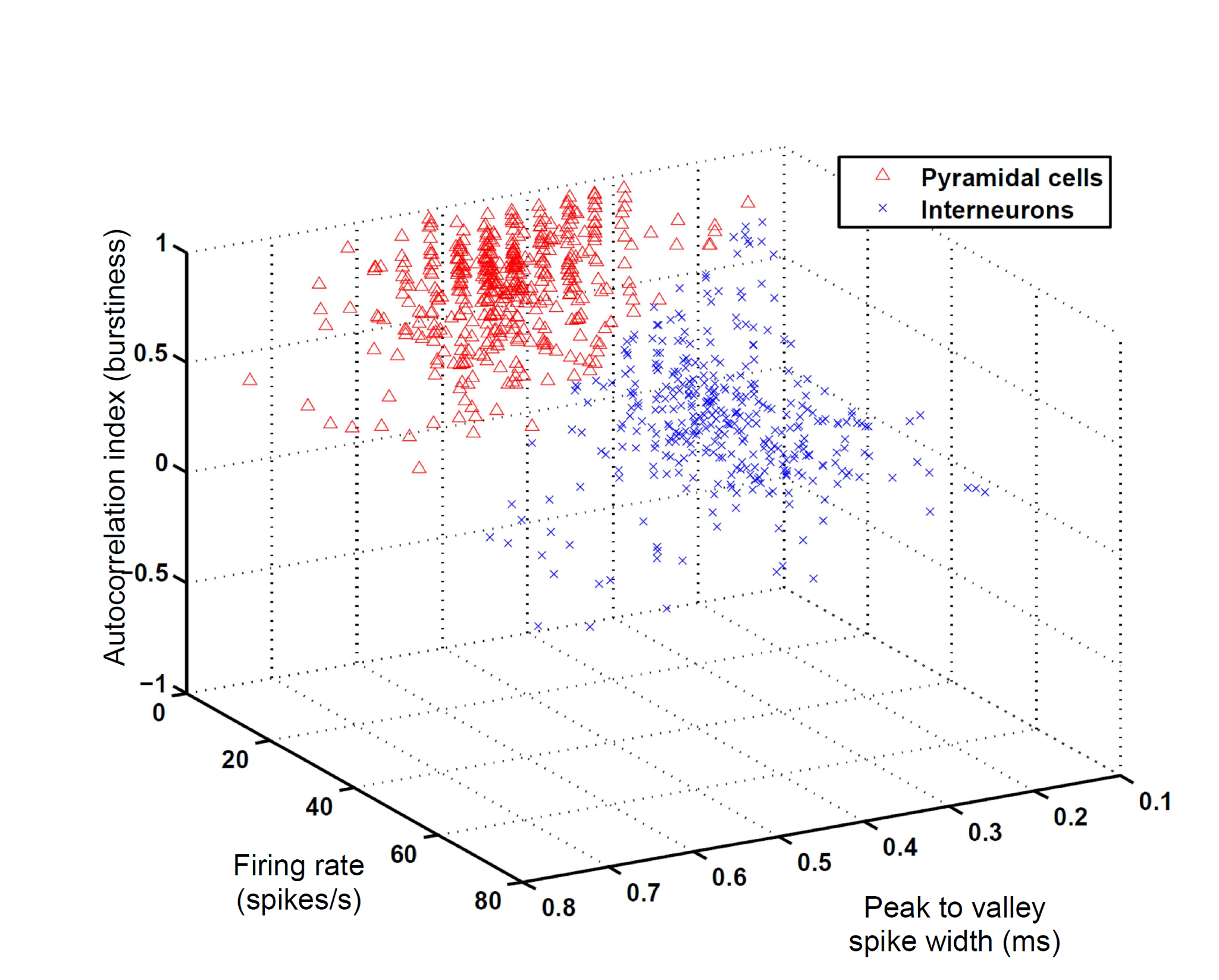

Supplement: Figure S2 — Separation between pyramidal cells and interneuron's. Three criteria were used to separate pyramidal cells: mean firing rate[51], [52], spike duration[53], and the autocorrelation function[54]. Firing rate was measured over the whole session. Spike duration was initially measured from peak-to-valley[3] and at 25% of maximum spike amplitude[54]; the former measure proved to be more reliable and was used in all sessions. The autocorrelation-derived index assessed the ratio of the difference between the number of spikes that occurred in a 2–5 ms post-spike window versus a 20–80 ms post-spike divided by the sum of the two. Spike duration and the autocorrelation-derived index yielded bimodal distributions. Based on firing rate, spike duration, and the autocorrelation-derived index, the complete set of neurons were clustered into 2 classes using a K-Means algorithm. Spike duration was longer in pyramidal cells (0.44±0.004 ms) compared to interneurons (0.25±0.0062 ms), consistent with[53] (interneurons' spike duration <0.3 ms, pyramidal cells' spike duration >0.3 ms)3. Putative pyramidal cells and putative interneurons had distinct autocorrelation profiles. Interneurons had significantly higher firing rate than pyramidal cells (13.06±0.68, and 2.63±0.16 spikes/s, respectively, Wilcoxon rank sum test p<0.000001). The firing rate of our pyramidal cells appears to be slightly higher than previously reported (e.g. 1.4±0.16 in[54]). We assume it is due to the fact that our apparatus was small and the majority of the recording was done during the task, so the probability of the discharge of a place cell would be higher compared to the standard task in which a rat runs in a larger maze. (TIF) [file pone.0016462.s004.tif]

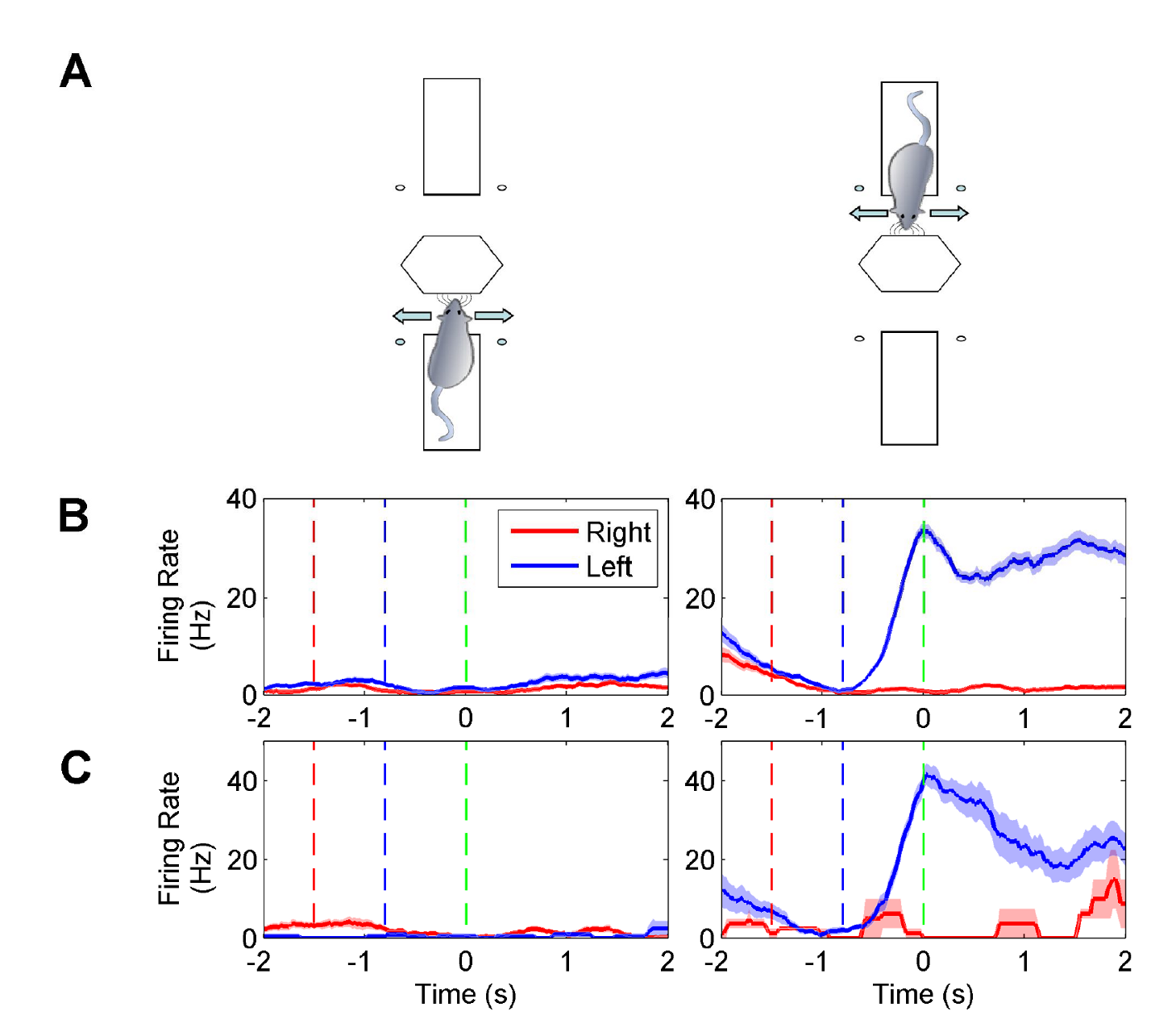

Supplement: Figure S3 — Firing of reward location neurons during incorrect trials. In our experiments, the number of error trials was low. For this reason, we were not able to obtain clear or stable statistical results on error trials. Nevertheless, in a few cases, there was a sufficient number of error trials, and with the same temporal rhythm, to allow spike times to be reliably aligned to behavior. For the neuron illustrated here, when the rat turned to the Left reward location in error (C, left column) the response profile closely resembled that present when the rat turned Left reward location on correct trials (B, left column). This indicates that the neuron's firing was correlated with the behavior of the rat. It does not specify, of course, which aspect of the behavior the neuron tracked – the perceived stimulus category, motor action, or the place. (A) Icon indicating the position of the animal in the behavioral setup. (B) Firing during correct trials, (C) firing during incorrect trials. (TIF) [file pone.0016462.s005.tif]

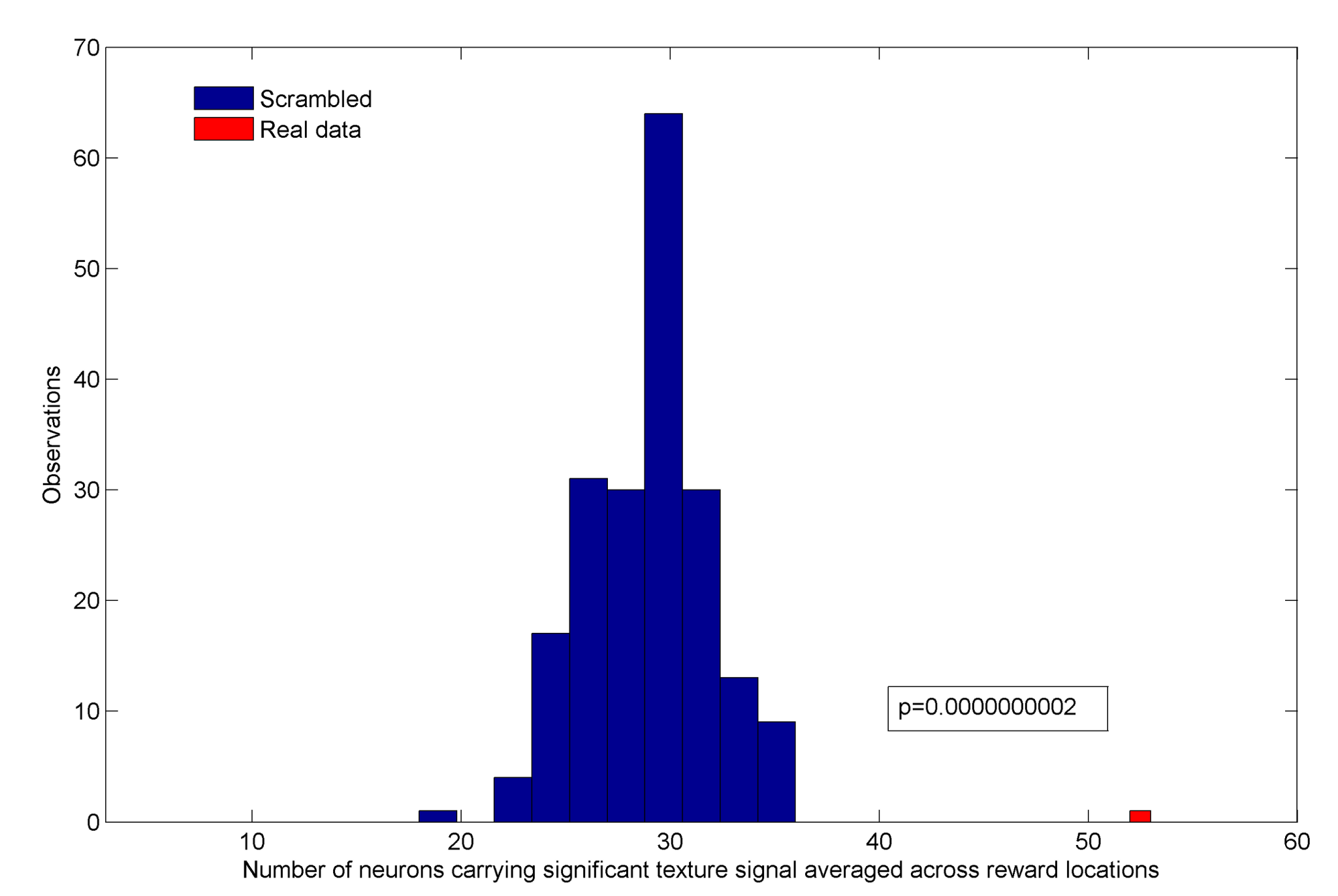

Supplement: Figure S4 — Texture neurons meta-analysis. To estimate the probability of finding the number of neurons with the significance threshold of p<0.05 we have performed a meta-analysis of the data. The distribution in blue represents the number of neurons which is expected to surpass our threshold of significance (scrambled) in averaged across all four reward location and the red bar indicates the real number of texture neurons on average associated with any given reward location in the real data. The p value of 0.0000000002 (z-test) indicates that it was highly unlikely to find such number of neurons that carry significant amount of texture information by chance. (TIF) [file pone.0016462.s006.tif]

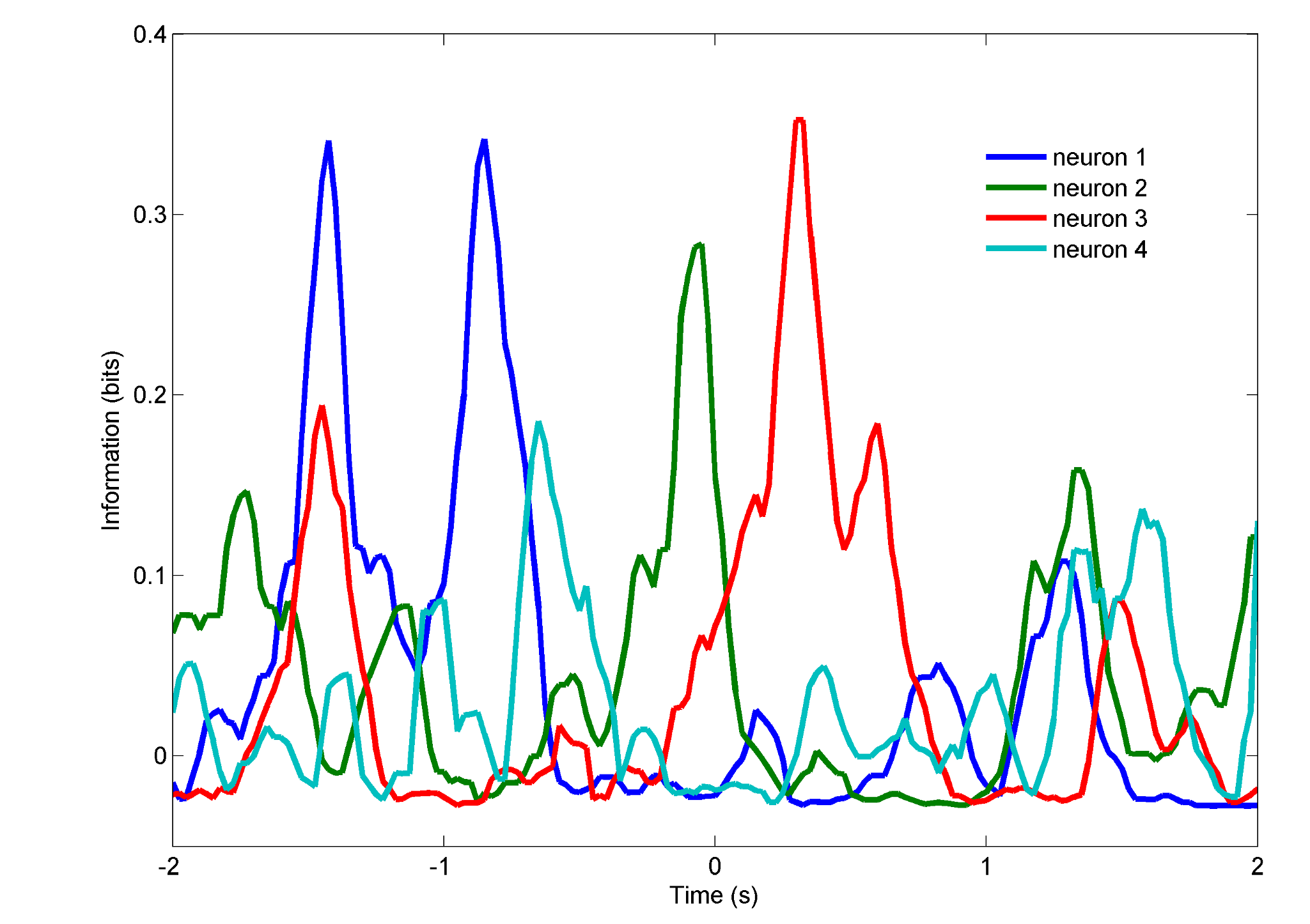

Supplement: Figure S5 — Information about texture is distributed across neurons over time. Information profiles of 4 simultaneously recorded neurons. Only traces of information associated with right turn on platform B are shown. Note that simultaneously recorded neurons carried information about texture at different points in time. Such a distribution of information was typical and led to Figure 5a when all traces were averaged. (TIF) [file pone.0016462.s007.tif]

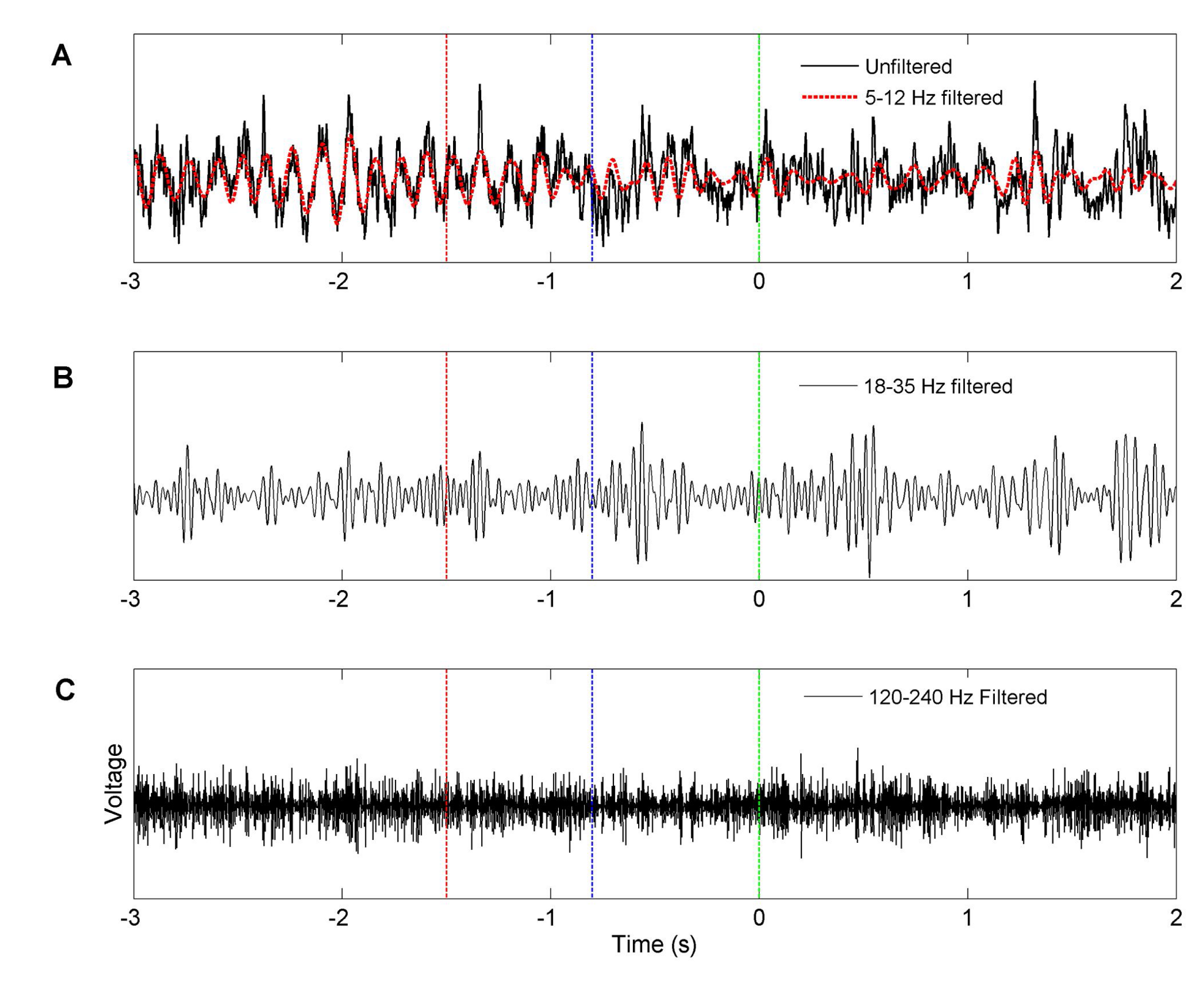

Supplement: Figure S6 — Reward-aligned local field potentials in a single trial. (A) Raw local field potential voltage trace (black) and 5–12 Hz (theta) filtered voltage trace (red). Red, blue, and green vertical dashed lines indicate the estimated start of time windows during which rats engaged in touch, turn and reward collection. (B) 18–35 Hz (beta and low gamma) band pass-filtered voltage trace. (C) 120–240 Hz band pass-filtered local voltage trace. (TIF) [file pone.0016462.s008.tif]

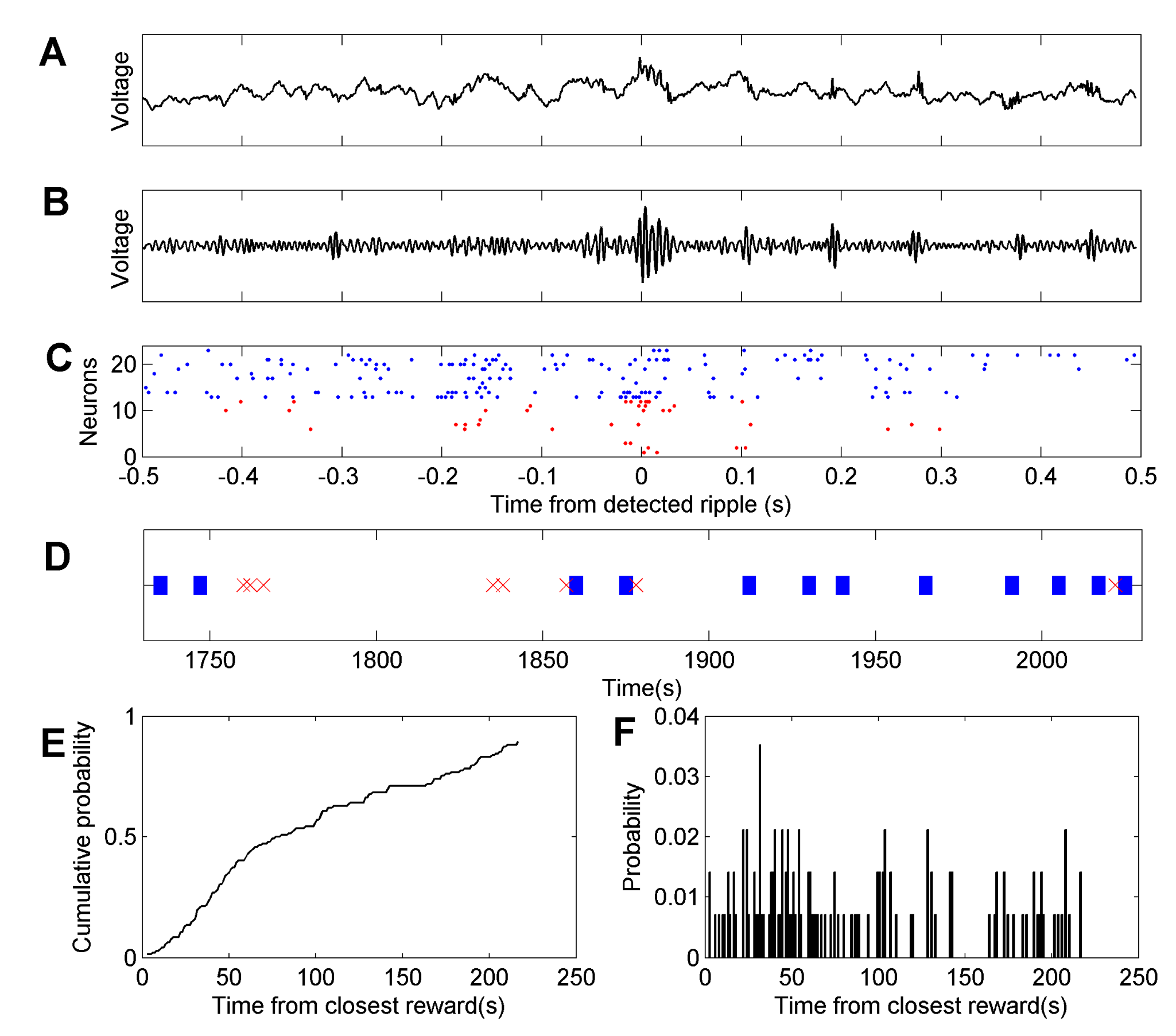

Supplement: Figure S7 — Ripple events. (A) Raw (unfiltered) local field potential voltage trace aligned on the maximum of the ripple event. (B) 120–240 Hz band pass filtered local field potential of the same voltage trace. (C) Raster plot of 23 simultaneously recorded neurons aligned on the ripple event. Red – pyramidal cells, blue – interneurons. (D) Relationship between ripple events (red X) and trials. Each blue rectangle represents a 4-second trial centered on the moment of reward onset. Ripples occurred most commonly when the rat took an extended pause within the session. (E) Cumulative probability of observing a ripple event as a function of distance in time from the closest reward delivery event. (F) Probability of observing a ripple event from the closest in time reward delivery event. Plots (E) and (F) indicate that the occurrence of a ripple event during the behavioral task was extremely rare. No cells were found to be active only during sharp waves (defined according to[55]). The cell whose activity was most attributable to sharp waves fired just 7% of its spikes during the waves. (TIF) [file pone.0016462.s009.tif]

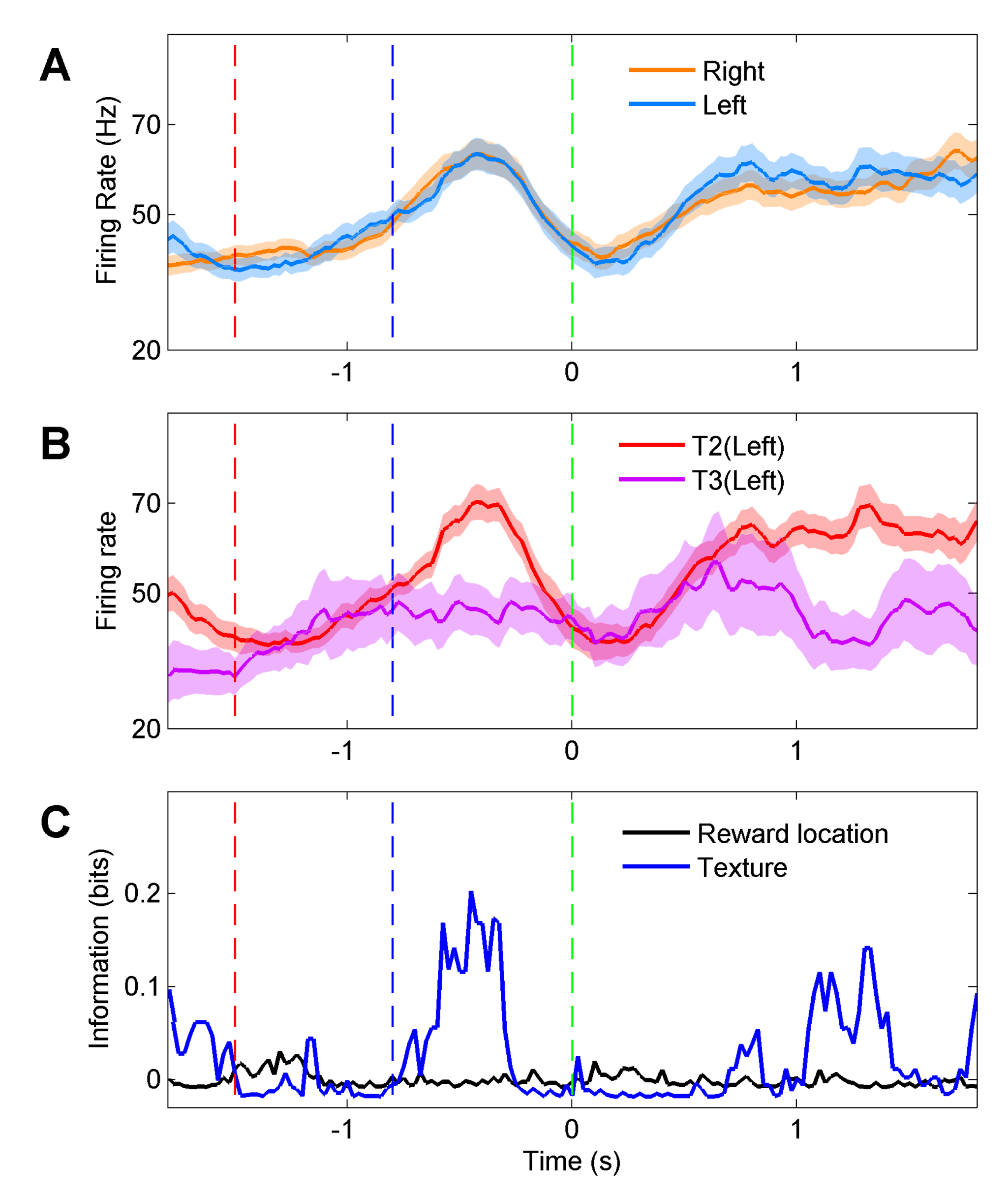

Supplement: Figure S8 — Representation of texture without reward location. Presence of a texture signal does not require the presence of a reward location signal. (A) The firing rates profiles associated with the animal's selection of the left and right reward locations were overlapping at all times on platform A. (B) Firing rate profiles associated with the left reward location on platform A are separated according to the texture present on each trial; now stimulus-specific activity can be discerned. (C) Temporal profile of information about reward location carried by neuronal firing rate; values were close to zero (black). In contrast, large quantities of texture information (blue) were present both during contact time and during reward collection. (TIF) [file pone.0016462.s010.tif]

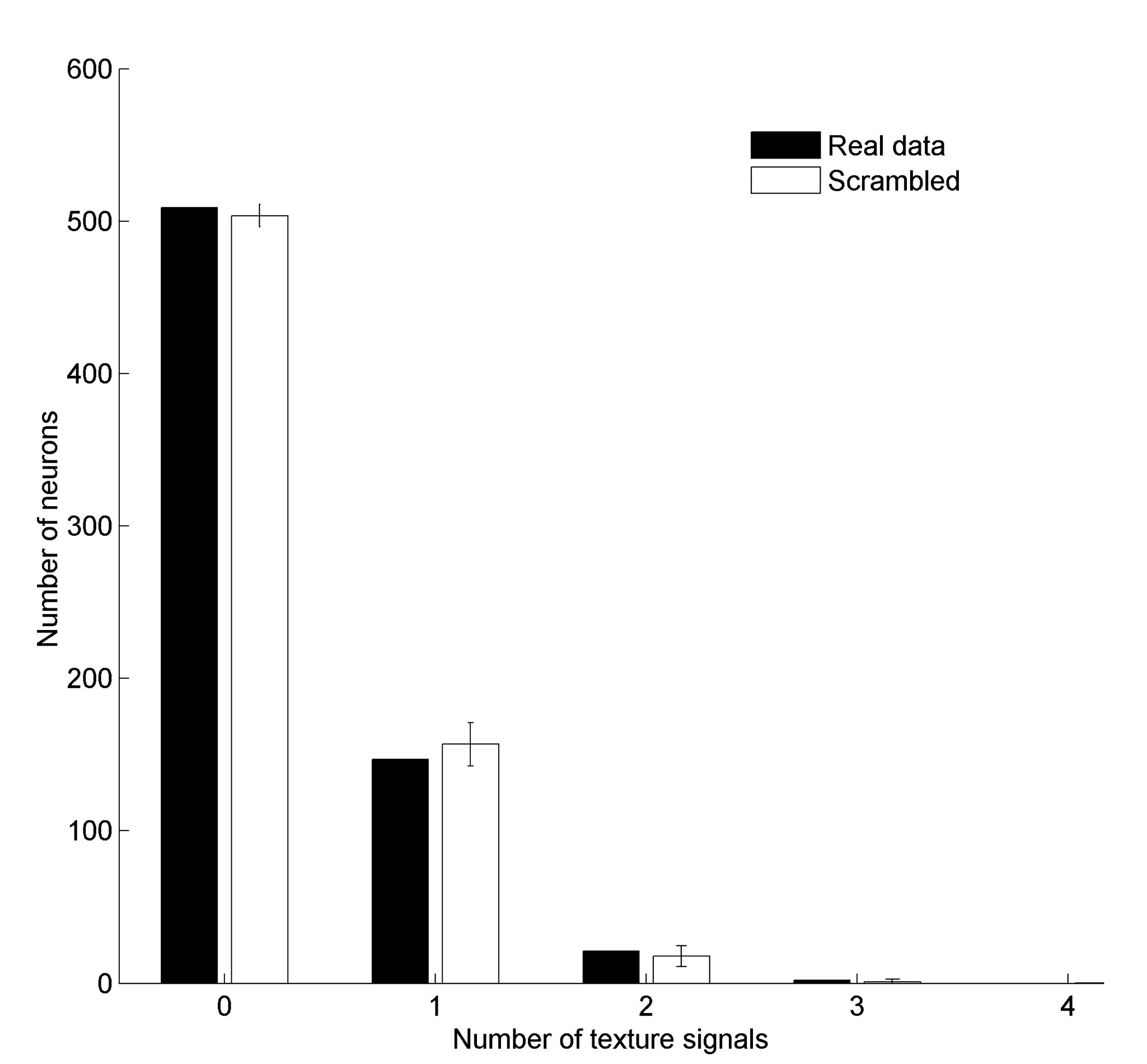

Supplement: Figure S9 — Independence of populations recruited to encode texture in different locations. In the two-platform task, neurons were tested for the presence of texture information on four occasions (the left and right texture pairs on platform A, and the left and right texture pairs on platform B). We asked whether the presence or absence of information about texture within one pair influenced the probability that a neuron would carry information about texture within any other texture pairs. Black bars indicate the numbers of neurons that carried a given number of texture signals, from 0 to 4. Of all 170 “texture neurons”, the majority (147) distinguished between only one texture pair on only one platform (for example, those neurons illustrated in Figure 3). Just 24 of 170 carried signals for more than one texture pair. The white bars indicate the count of neurons that would be expected to carry a given number of texture signals if encoding of each texture pair were independent, with 95% confidence intervals included. The simulated and observed distributions are closely matched. We speculate that the independence of the tactile representations on the two platforms was an outcome of the rats' interpretation of the platforms as two independent contexts; they needed to be retrained on the second platform and showed consistently lower performance there (see Touch-guided Behavior). We suggest that the hippocampal representation of salient events underwent a reset each time the rat was moved between platforms. (TIF) [file pone.0016462.s011.tif]

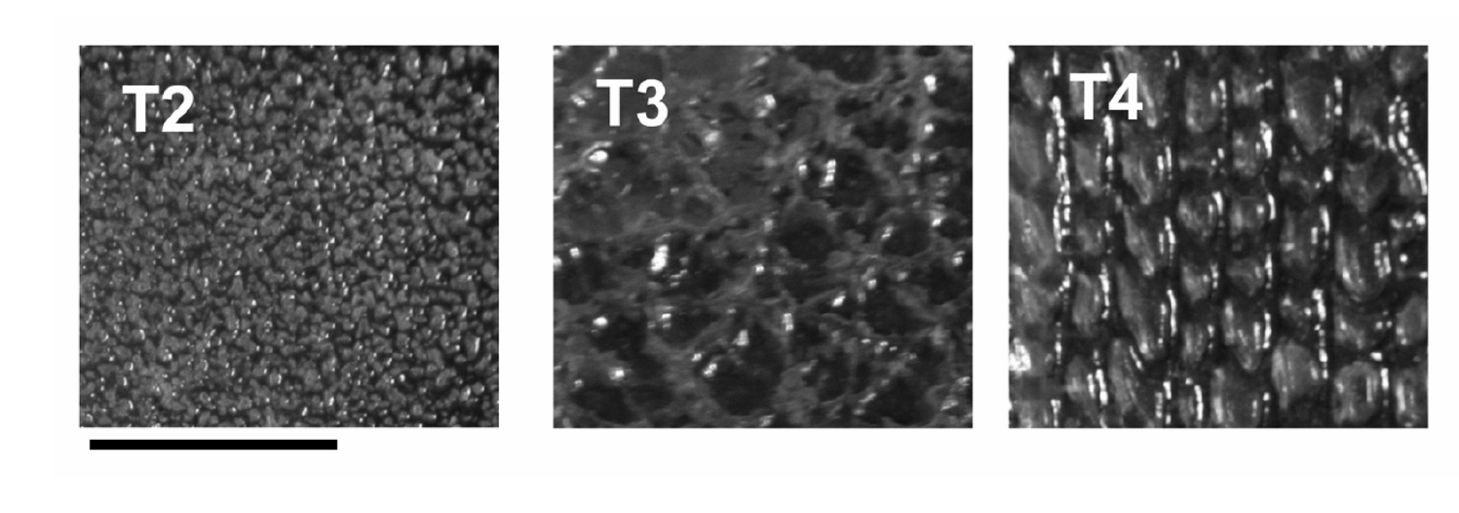

Supplement: Figure S10 — Tactile stimuli. Photographs of rough tactile discrimanda, textures 2–4 (T2–T4). T1 was a smooth plate. Scale bar is 10 mm. (TIF) [file pone.0016462.s012.tif]
